# Machine learning to attribute the source of *Campylobacter* infections in the United States: A retrospective analysis of national surveillance data

**DOI:** 10.1016/j.jinf.2024.106265

**Published:** 2024-09-07

**Authors:** Ben Pascoe, Georgina Futcher, Johan Pensar, Sion C. Bayliss, Evangelos Mourkas, Jessica K. Calland, Matthew D. Hitchings, Lavin A. Joseph, Charlotte G. Lane, Tiffany Greenlee, Nicolas Arning, Daniel J. Wilson, Keith A. Jolley, Jukka Corander, Martin C.J. Maiden, Craig T. Parker, Kerry K. Cooper, Erica B. Rose, Kelli Hiett, Beau B. Bruce, Samuel K. Sheppard

**Affiliations:** a Ineos Oxford Institute for Antimicrobial Research, Department of Biology, University of Oxford, Oxford, United Kingdom; b The Milner Centre for Evolution, Department of Biology and Biochemistry, University of Bath, Claverton Down, Bath, United Kingdom; c Department of Mathematics, University of Oslo, Oslo, Norway; d Bristol Veterinary School, University of Bristol, Langford, Bristol, United Kingdom; e Zoonosis Science Centre, Department of Medical Sciences, Uppsala University, Uppsala, Sweden; f Oslo University Hospital, Oslo Centre for Biostatistics and Epidemiology, Oslo, Norway; g Swansea University Medical School, Swansea University, Swansea, United Kingdom; h Division of Foodborne, Waterborne, and Environmental Diseases, Centers for Disease Control and Prevention, Atlanta, GA, USA; i Center for Food Safety and Applied Nutrition, Food and Drug Administration, College Park, MD, USA; j Big Data Institute, Oxford Population Health, University of Oxford, Li Ka Shing Centre for Health Information and Discovery, Old Road Campus, Oxford, United Kingdom; k Department for Continuing Education, University of Oxford, United Kingdom; l Department of Biology, University of Oxford, Oxford, United Kingdom; m Department of Mathematics and Statistics, University of Helsinki, Helsinki, Finland; n Parasites and Microbes, Wellcome Sanger Institute, Cambridge, United Kingdom; o Produce Safety and Microbiology Research Unit, Agricultural Research Service, US Department of Agriculture, Albany, CA, USA; p School of Animal and Comparative Biomedical Sciences, University of Arizona, Tucson, AZ, USA; q Center for Food Safety and Applied Nutrition, Food and Drug Administration, Laurel, MD, USA

**Keywords:** Campylobacteriosis, Gastroenteritis, Source attribution, Machine learning, Chicken consumption

## Abstract

**Objectives:**

Integrating pathogen genomic surveillance with bioinformatics can enhance public health responses by identifying risk and guiding interventions. This study focusses on the two predominant *Campylobacter* species, which are commonly found in the gut of birds and mammals and often infect humans via contaminated food. Rising incidence and antimicrobial resistance (AMR) are a global concern, and there is an urgent need to quantify the main routes to human infection.

**Methods:**

During routine US national surveillance (2009–2019), 8856 *Campylobacter* genomes from human infections and 16,703 from possible sources were sequenced. Using machine learning and probabilistic models, we target genetic variation associated with host adaptation to attribute the source of human infections and estimate the importance of different disease reservoirs.

**Results:**

Poultry was identified as the primary source of human infections, responsible for an estimated 68% of cases, followed by cattle (28%), and only a small contribution from wild birds (3%) and pork sources (1%). There was also evidence of an increase in multidrug resistance, particularly among isolates attributed to chickens.

**Conclusions:**

National surveillance and source attribution can guide policy, and our study suggests that interventions targeting poultry will yield the greatest reductions in campylobacteriosis and spread of AMR in the US.

**Data availability:**

All sequence reads were uploaded and shared on NCBI’s Sequence Read Archive (SRA) associated with BioProjects; PRJNA239251 (CDC / PulseNet surveillance), PRJNA287430 (FSIS surveillance), PRJNA292668 & PRJNA292664 (NARMS) and PRJNA258022 (FDA surveillance). Publicly available genomes, including reference genomes and isolates sampled worldwide from wild birds are associated with BioProject accessions: PRJNA176480, PRJNA177352, PRJNA342755, PRJNA345429, PRJNA312235, PRJNA415188, PRJNA524300, PRJNA528879, PRJNA529798, PRJNA575343, PRJNA524315 and PRJNA689604. Contiguous assemblies of all genome sequences compared are available at Mendeley data (assembled *C. coli* genomes doi: 10.17632/gxswjvxyh3.1; assembled *C. jejuni* genomes doi: 10.17632/6ngsz3dtbd.1) and individual project and accession numbers can be found in Supplementary tables S1 and S2, which also includes pubMLST identifiers for assembled genomes. Figshare (10.6084/m9.figshare.20279928). Interactive phylogenies are hosted on microreact separately for *C. jejuni* (https://microreact.org/project/pascoe-us-cjejuni) and *C. coli* (https://microreact.org/project/pascoe-us-ccoli).

## Introduction

*Campylobacter* is the most common cause of bacterial gastro-intestinal infections in high-income countries including the United States. Currently, the US Centers for Disease Control and Prevention (CDC) estimates approximately 17 cases per 100,000 people, equating to 1.5 million illnesses each year with many more un-diagnosed or unreported cases.^1,2^ There are differences in disease reporting, based on bacterial culturing and culture-independent methods,^3^ and many more cases go unreported as symptoms, including nausea, diarrhoea, and vomiting, typically subside within five days. However, over 13,000 cases annually require hospitalisation, of which around 1% are fatal.^4^ Occasional persistent and debilitating sequelae, such as reactive arthritis, Guillian-Barré and irritable bowel syndromes intensify the public health impact.^5,6^ In serious cases, infections may require treatment with antibiotics. However, as for some other gastrointestinal pathogens, antimicrobial resistance (AMR) is increasing,^7–10^ limiting treatment options. Control of multidrug resistant (MDR) *C. jejuni* is now considered a high priority by the World Health Organization (WHO),^11^ and there is an urgent need to identify the relative contribution to human infection of different sources of this pervasive foodborne pathogen.

Understanding the origin of human campylobacteriosis is challenging. The ubiquity of *C. jejuni* and *C. coli* in animal intestines, and its shedding into the environment, leads to numerous infection routes. Recent confirmed outbreaks have been traced to contaminated dairy products,^12^ contact with pets,^13–15^ chicken livers,^16–19^ raw peas (wild birds),^20^ and shellfish,^21^ but these represent only a small number of cases.^22^ Most human infections are sporadic, likely representing a continuous stream of contamination from diffuse sources.^22–26^ Indirect evidence of infection sources has come from risk assessments and case-control studies,^27–29^ but DNA-sequence-based methods have provided opportunities for promising novel approaches. *Campylobacter* has adapted to colonise multiple host species, leaving signatures in the genome. Thus, the origin of isolates that infect humans can be determined by analysing the clinical isolate genomes. Source attribution studies have been employed to identify poultry as the primary source of infections in several European countries.^24,26,30–32^ However, the importance of different disease sources varies among countries and regions,^23,33–35^ and there are currently no reliable, routine source attribution estimates for the US.

Public health organizations in the US have been among the fastest to incorporate genome sequencing technologies into routine national surveillance.^36^ Cross-agency data sharing, including *Campylobacter* genomes from clinical infections and host sources, is ideal for quantitative source attribution modelling to determine the relative contribution of different infection sources from genotype data. In this study, we analysed *Campylobacter* genomes from US national surveillance programs using machine learning and probabilistic source attribution to estimate the sources of human *C. jejuni* and *C. coli* isolates since 2009. *Campylobacter* infection can be considered through a “one health” perspective, where rapid intensification in agricultural practices has contributed to the evolution of the pathogen^37^ and widespread use of antibiotics has been selected for increased rates of antibiotic resistance.^38,39^ Increasing numbers of human infections can be attributed to MDR isolates from poultry sources. This represents a significant public health risk and provides important information for interventions and infection control.

## Methods

### Sampling and microbiology

Human clinical campylobacteriosis cases in the US have been nationally notifiable since 2015, and the CDC has supported collection and sequencing of ~9000 *Campylobacter* isolates since 2009 (Supplementary tables S1 & S2). Our dataset included *C. jejuni* and *C. coli* isolates collected from confirmed campylobacteriosis cases, and historical cases reported nationally by state and local public health laboratories. These were grouped within 10 US health and human services (HHS) regions to de-identify the source state (Supplementary Fig. S1). Following the removal of potential duplicate samples, the collection comprised 8856 genomes. No ethical approval was required as all isolates were collected as part of routine diagnosis and surveillance.

Context isolates were collected from livestock animals contemporaneously by three US public health agencies. These were supplemented with a previously published global collection of genomes from wild birds^40^ and environmental isolates.^41^ Genomic signatures of host source are robust to spatial and temporal sample variation in *Campylobacter*,^42^ therefore isolate genomes collected at different times from multiple countries can be used in source attribution models. This strong host signal can be observed among black birds, gulls, sandpipers/dunlins, and ducks/geese from Europe and Australia that are found together in discrete phylogenetic clusters, separate from livestock,^37^ despite the considerable distances between samples.^43^

The Food Safety and Inspection Service (FSIS) is part of the national system to monitor the effectiveness of controls to reduce *Campylobacter* and other foodborne pathogens in raw meat and poultry products. As part of its role in routine surveillance, FSIS has employed whole genome sequencing to identify pathogens of concern in poultry products since 2013 (specifically, *Campylobacter* since 2019) (Supplementary table S2). Sampling was conducted across the US, including from animal faeces and food. FSIS USDA sampled chicken, turkey, cattle, and swine and animal food products at slaughter and processing facilities. Animal ceca were sampled immediately after slaughter. Turkey carcasses were swabbed; chicken carcasses and parts were rinsed, while portions of ground chicken and turkey and retail meat were collected for testing. *Campylobacter* was isolated using direct plating or enrichment using methods as described in the USDA’s Microbiology Laboratory Guidebook Chapter 41.

Retail poultry isolates were included in the study from the NARMS (National Antimicrobial Resistance Monitoring System) retail meat surveillance program at the Food and Drug Administration’s (FDA) Center for Veterinary Medicine (CVM). FDA collaborates with state public health and academic partners to collect and test retail poultry products. Isolation of *Campylobacter* from poultry samples was performed at participating laboratories using methods outlined in the NARMS Manual of Laboratory Methods, Retail Meat Isolation Protocol. Whole genome sequencing of isolates was conducted, and sequence data were uploaded to NCBI. Species confirmation and antimicrobial susceptibility testing were then performed at FDA laboratories.

### Genome sequencing, assembly, and quality control

Sequencing was performed locally by laboratories with standard CDC operating procedures for *Campylobacter*, using Illumina sequencers (San Diego, CA).^44^ Sequence reads were downloaded and assembled using FLASH (version 1.2.11)^45^ and SPAdes (version 3.12.0)^46^ (accession numbers in Supplementary tables S1 & S2). Assemblies were annotated using prokka (version 1.13).^47^ Quality control statistics were generated using QUAST (version 4.6.3),^48^ fastqc (version 0.11.7)^49^ and snippy (version 3.2).^50^ Non-*C*. *jejuni* or *C. coli* genomes were removed, as were assemblies comprising > 500 contigs, or an assembly length < 1.5 Mbp or > 2 Mbp. Datasets also included *C. jejuni* (NCTC 11168) and *C. coli* (ASM202418v1) reference strains.^35,51^ Datasets were augmented with additional *C*. *jejuni* and *C*. *coli* isolates from previous studies,^37,52^ subject to the same selection criteria. The final dataset comprised 18,306 *C. jejuni* isolates from humans, chickens, cows, pigs, and turkeys; and 7253 *C. coli* isolates from humans, cows, chickens, and wild birds (Supplementary Fig. S2;). Phylogenies were constructed using Mashtree (version 1.0.4),^53^ visualised and shared on microreact.^54^ Each isolate was assigned sequence types (STs) using the seven-locus MLST scheme incorporated in pubMLST^55,56^ (Supplementary tables S1 & S2; Supplementary Fig. S3). Isolates that shared five or more MLST alleles were grouped into defined clonal complexes (CCs)^57,58^ or *C*. *coli* clades and clustered on the phylogeny with previously typed isolates.^59^

### Random forest identification of pangenome host segregating markers between reservoir sources

Isolates were grouped into food categories according to the Interagency Food Safety Analytics Collaboration (IFSAC) categories designed for harmonisation among public health agencies.^60,61^ *C*. *jejuni* isolates from chicken, cattle, and wild bird sources, and *C. coli* isolates from chicken, cattle, pork, and turkey sources were included in separate, species-specific random forest analyses (Supplementary Fig. S2).

Unitigs were generated from assemblies using unitig counter (version 1.1.0),^62^ and unitigs found in the same subset of isolates were grouped into a single presence-absence pattern.^63^ Pattern distribution was scored using mutual information (MI) in each reservoir source population.^64^ Statistically, MI measures the marginal association between the two variables. Biologically, it gives a measure of how effectively a pattern (and ultimately unitigs) can distinguish the source in question from every other source, where greater values represent a greater separation between the source and every other source. Patterns with the greatest source-associated MI score (100,000 per source) were chosen for machine learning (ML) attribution analysis. Previous ensemble ML studies using *Campylobacter* genomes have identified random forest analyses, with XGBoost on a balanced cgMLST dataset, as an optimal approach for source attribution.^65^ A weighted random forest using equal numbers of isolates from each source population was generated using the R package ranger (version 0.12.1)^66^ and repeated 10 times. Each model was trained to predict source populations using patterns and 10,000 bootstrap trees were trained, and out-of-bag predictions calculated. The robustness of each model was tested via recalculation using 10 different seeds.

A pangenome was constructed from a subset of isolates. To ensure that these represented diversity across *C. jejuni* and *C. coli*, unitig covariation patterns were determined for all isolates and 2000 isolates were chosen per source. These included covariation patterns with the highest MI between sources, such that all selected patterns were present in at least 20 isolates. Reference strains for each species were also added and a pangenome was constructed using PIRATE (version 1.0.4)^67^ with the option to generate pangenome alignments. Unitigs were mapped to the pangenome alignment using bwa fastmap (version 0.7.17),^68^ with the maximum interval size set to a number greater than the number of isolates and the position of every match was returned. Full length matches at unique positions that did not border different genes were analysed and the number of hits and highest MI score was recorded for each matched unitig in each source population. Five genes with the greatest number of matches and five genes with the greatest MI scores per source population were carried forward as host-segregating markers.

### Probabilistic attribution of the source of human infection isolates

Source attribution models were employed to predict the most likely source host for each human campylobacteriosis case. Allele numbers were assigned to isolate genomes for each of the host segregating markers (Supplementary table S3) and compared with the current best performing core genome multi-locus sequence typing (MLST) method for source attribution. Missing alleles, novel alleles, and unique combinations of multicopy alleles were each given a typing number starting at 10,000. *C. coli* isolates from turkey and chicken sources were merged into a single source (poultry) for attribution analyses with iSource, as there was a significant overlap between markers that could accurately segregate the sources.^24,32^ The iSource algorithm was run with a thinning interval of 1 for 100,000 iterations, reporting every 50 iterations. 10 runs of self-attribution were performed for each species, masking the source of one-third of isolates from non-clinical populations. Human clinical isolates were then attributed separately for *C. jenuni* and *C. coli* and compared across health regions and putative sources.

### Comparison with core genome MLST method for source attribution

To further verify our findings, the putative source of human infections was also attributed using cgMLST profiles and implementing machine learning algorithms in aiSource.^65^ Profiles were exported from PubMLST using their cgMLST loci (*C. jejuni-C*.*coli cgMLST v1*.*0*).^55,69^ Incomplete or missing alleles were replaced by the flag “NA” and treated as a distinct allele using the catboost algorithm, which can incorporate categorical data. No under-sampling or hyperparameter optimisation was used in training the model. A verification dataset was constructed of human *C. coli* (n = 696), human *C. jejuni* (n = 8160), non-human *C. coli* (n = 6538) and non-human *C. jejuni* (n = 10,187) isolates. Non-human *C. coli* isolates sampled from environmental or wild bird sources were grouped as “other”, resulting in a dataset comprising chicken (n = 3755), cattle (n = 1181), pig (n = 1112), turkey (n = 398) and other (n = 91) isolates. *C. jejuni* isolates sampled from environmental, turkey or pig sources were grouped as “other”, resulting in a dataset comprising chicken (n = 5660), cattle (n = 3842), wild birds (n = 328) and other (n = 356). All scripts are available on GitHub (https://github.com/narning1992/aiSource).

### Identification of antimicrobial resistance elements

Nucleotide sequences for all genomes were screened using StarAMR (version 0.4.0)^70^ for the presence of known AMR determinants against ResFinder^71^ and point mutations with PointFinder (databases updated April 2020).^72^ A positive hit was defined when a gene had > 75% nucleotide identity over > 50% of the sequence length. A matrix of gene presence for every antimicrobial resistance gene was generated for every genome. Comparisons were made between species, sampled sources, and assigned sources following attribution.

## Results

**Fig. 1:**
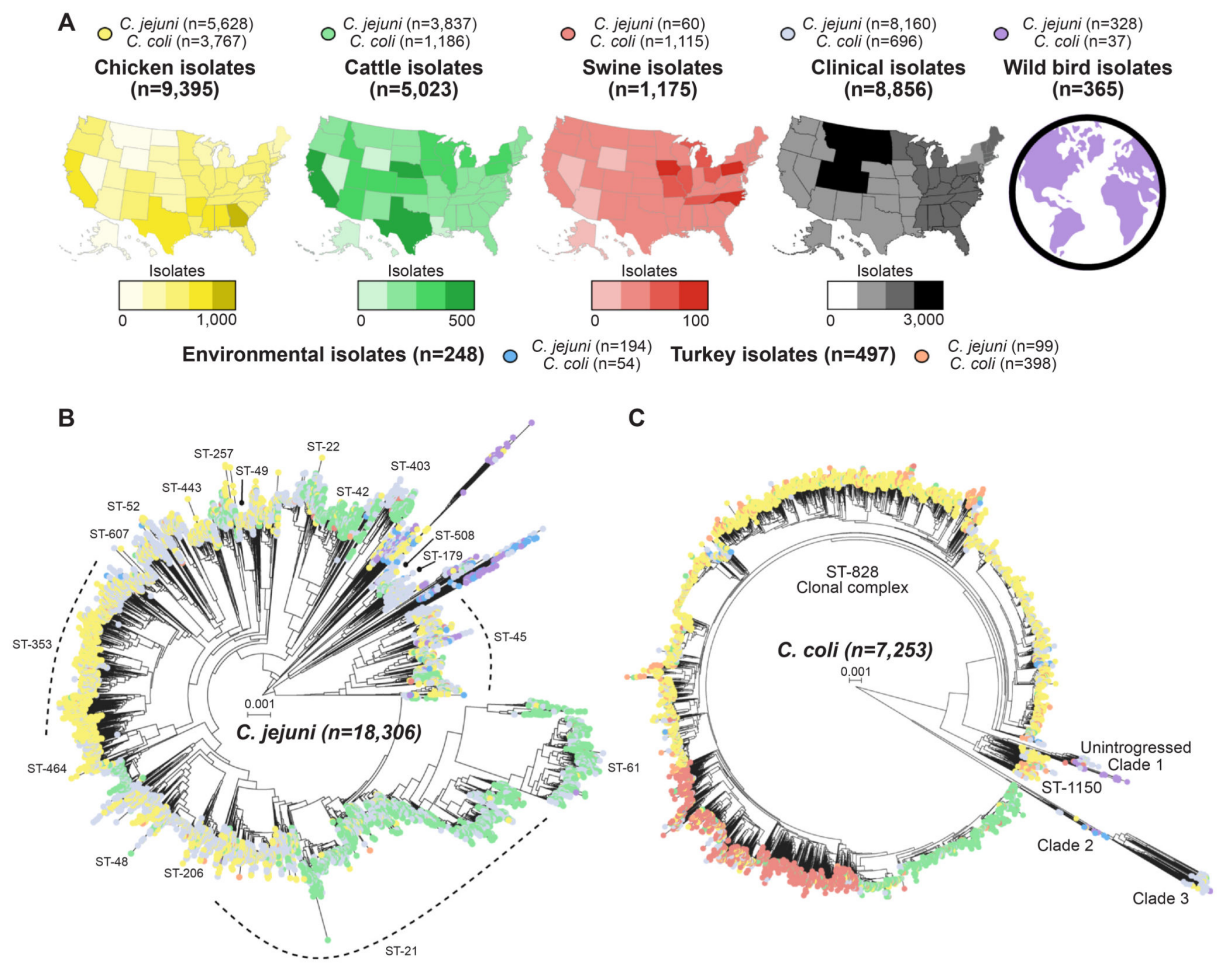
Isolate genomes used in this study. **(A)** Overview of the number of isolates from each source, including chicken (yellow; n = 9395), cattle (green; n = 5023), swine (pink; n = 1175), clinical cases (grey; n = 8856), environmental sources (blue; n = 248), turkey (orange; n = 497) and a global collection from birds (purple; n = 365). Samples were collected from all over the US, with states that contributed higher numbers of samples coloured more deeply on the maps. **(B)** Phylogeny of 18,306 *C. jejuni* genomes constructed using mashtree, coloured by source. The two large host generalist clonal complexes (ST-21 CC and ST-45 CC) are labelled along with the chicken-associated clonal complex.^74^ **(C)** Mash phylogeny of 7253 *C. coli* coloured by source. The large host generalist clonal complex, ST-828 CC and three ancestral clades (Clades 1, 2 and 3) are labelled along with the highly introgressed ST-1150 clonal complex.^41,58,75^

In total, 8856 isolates were collected and sequenced from confirmed human campylobacteriosis between 2009–2019, with most identified as *C. jejuni* (n = 8160; 92%; Supplementary table S1). The remaining isolates were predominantly *C. coli* (n = 696; 8%), consistent with the relative prevalence of species in similar surveillance studies from other high-income countries.^73^ Contemporaneous isolates were also collected and sequenced by the CDC, FSIS, and the FDA during this period from possible *Campylobacter* infection sources (n = 16,703; Supplementary table S2). These source data comprise *C. jejuni* (n = 10,146) and *C. coli* (n = 6557) isolates from chicken (n = 9395), cattle (n = 5023), pork (n = 1175), turkey (n = 497), wild bird (n = 365) and environmental (n = 248) sources (Fig. 1A).

### Population structure reveals host-associated C. jejuni and C. coli lineages

Clusters of isolates from the same host were clearly visible on *C. jejuni* and *C. coli* phylogenies (Fig. 1B, 1C), however there were differences between species. Common *C. jejuni* clonal complexes (CCs) included source-associated sub-lineages (Fig. 1B). CCs containing 10 or more human clinical isolates were analysed (Supplementary Fig. S3). There was evidence of cattle- and chicken-associated sub-lineage clustering within the ST-21 CC, one of the most common lineages found in human infections worldwide. The major chicken-associated clade contained many lineages where non-human samples were only collected from chickens, including the ST-464, ST-353, ST-574, ST-607, ST-52, ST-460, ST-443 and ST-354 CCs, although the ST-257 CC also included isolates from cattle. Cattle-associated lineages included the ST-22, ST-42, ST-403 and ST-508 CCs. Wild bird isolates also displayed strong clustering within the ST-692, ST-1287, ST-179, ST-177, ST-1034 and ST-1332 CCs. Clonal complexes containing multiple turkey isolates, such as the ST-179 and ST-692 CCs, also contained wild bird isolates. Environmental isolates also clustered with wild bird isolates in the ST-177, ST-1034 and ST-1332 CCs although the ST-574 CC contained both environmental and chicken isolates. In contrast to *C. jejuni*, most *C. coli* isolates belonged to a single clonal complex, the ST-828 CC. Within this clonal complex, isolates clearly segregated by host source (Fig. 1C).

### Identification of host segregating markers in C. jejuni and C. coli pangenomes

**Fig. 2:**
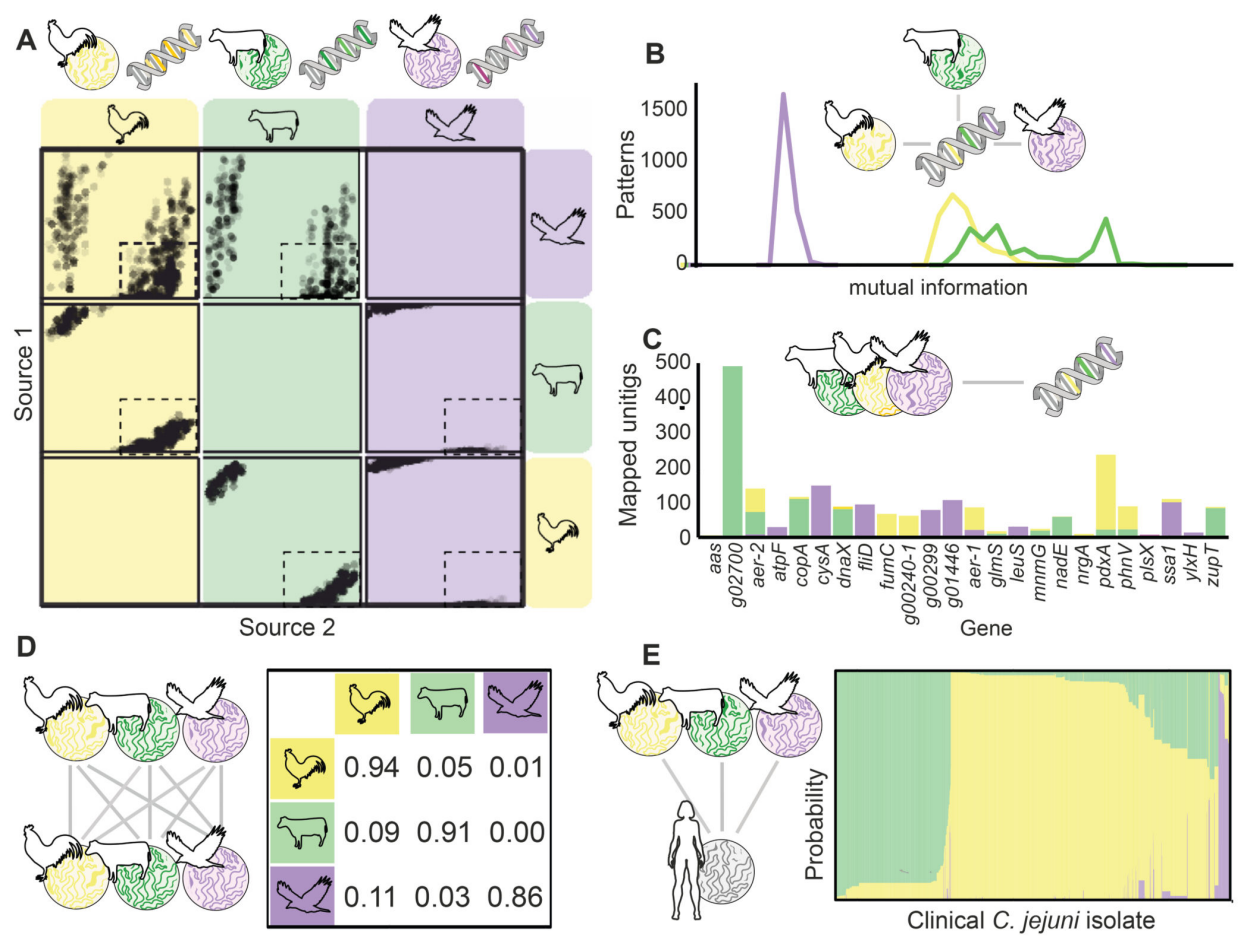
Overview of *C. jejuni* attribution study. **(A)** Random Forest analyses were performed on training data from chicken, cattle, and wild bird sources to score patterns of unitigs according to their ability to predict isolate source. Dots within each box show how common each pattern is in the two intersecting hosts. Patterns within the small, dotted boxes are common in the host on the horizontal axis, but rare in the host labelled on the vertical axis. **(B)** Patterns of unitigs with highly discriminatory mutual information (MI) scores were used to select markers for different hosts. **(C)** Genes from which unitig markers were selected and assigned allele numbers for attribution. **(D)** Markers were tested on a subset of the data for accuracy (overall accuracy > 90%); and **(E)** used to predict the source of 8160 *C. jejuni* infection cases.

**Fig. 3:**
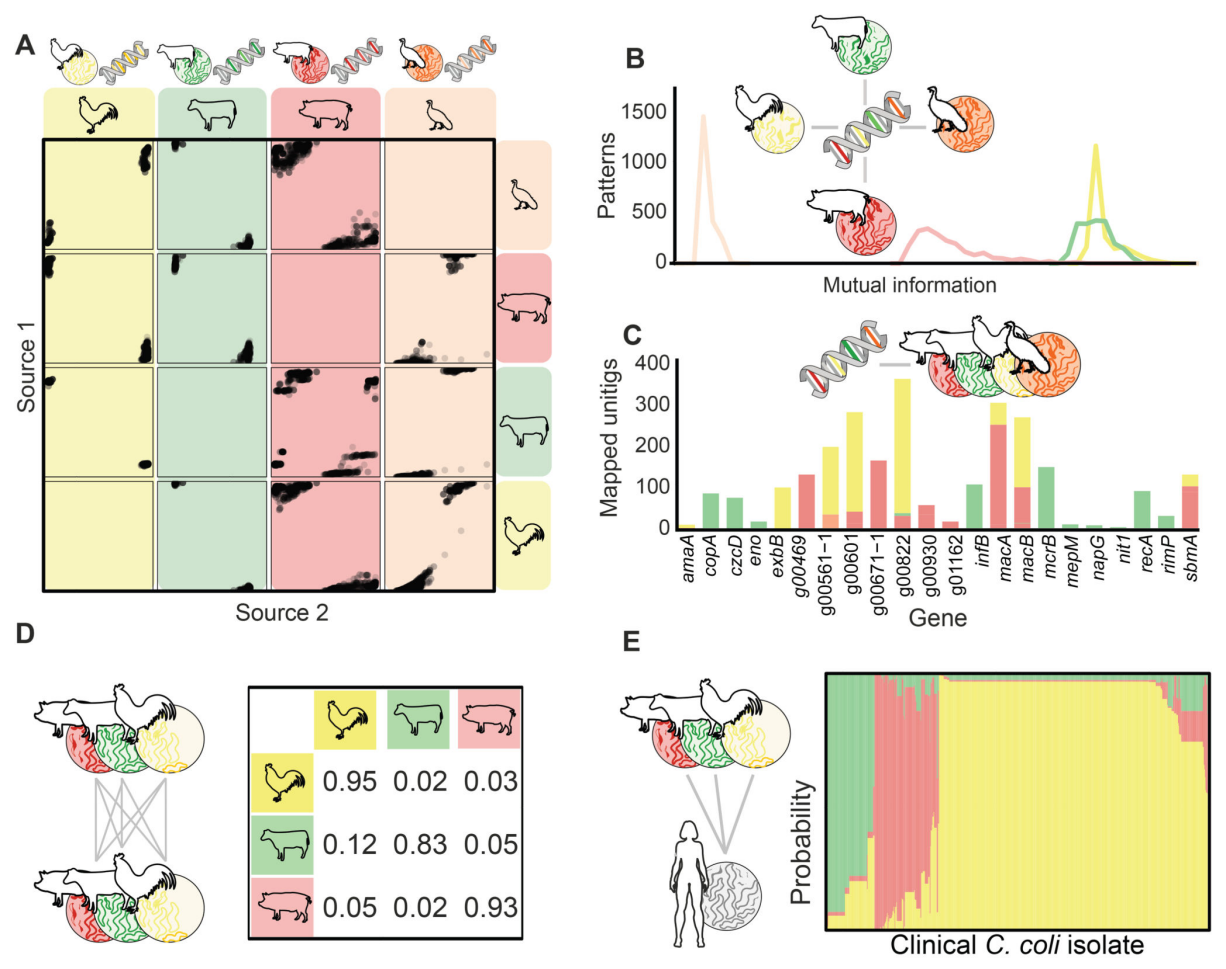
Overview of *C. coli* attribution study. **(A)** Random Forest analyses were performed on training data from chicken, cattle, turkey, and pig sources to score patterns of unitigs according to their ability to predict isolate source. Dots within each box show how common each pattern is in the two intersecting hosts. Patterns within the small, dotted boxes are common in the host on the horizontal axis, but rare in the host labelled on the vertical axis. Patterns of unitigs with highly discriminatory mutual information (MI) scores were used to select markers for different hosts. **(C)** Genes from which unitig markers were selected and assigned allele numbers for attribution. **(D)** Markers were tested on a subset of the data for accuracy (overall accuracy > 90%). **(E)** Chicken and turkey markers were combined to predict the source of 696 *C. coli* infection cases from poultry, cattle, and pigs.

Contigs from assembled *C. jejuni* and *C. coli* genomes were partitioned into 5,653,690 unitigs capturing variation in the core and accessory genomes. These were collapsed to groups of unitigs with shared presence-absence patterns (n = 3,580,741). Random forests were generated for *C. jejuni* (Fig. 2), and *C. coli* (Fig. 3) isolates using 298,330 and 350,575 source-associated patterns and scored for accuracy using mutual information (MI). There was much clearer partitioning between patterns that identified *C. jejuni* isolates from cattle and chicken, than those that identified wild bird isolates (Fig. 2A, B). Chicken and cattle patterns also demonstrated the highest discriminatory power (MI score) to identify potential *C. coli* sources (Fig. 3A, B). A pangenome was constructed which included the 2000 best performing patterns per potential source population, to which unitigs were mapped and the loci (genes) within identified. To improve computational runtime and develop a schema for public health use, the 30 most accurate unitigs were selected for each species, which encompassed 24 *C. jejuni* and 22 *C. coli* genes (Figs. 2C & 3C; Supplementary table S3).

### Chicken is the dominant source of US human infections

Isolates of each species from our clinical and attribution datasets were typed and assigned allele values for each of the selected host segregating markers (unitigs). Accuracy in identifying the correct source of an isolate was tested by masking the source of a random third of isolates from the training dataset (isolates from known sources) (Figs. 2D & 3D). Using an asymmetric island model (iSource^32^), 10 self-attribution tests were performed and averaged. The overall attribution accuracy was 90% for both *C. jejuni* and *C. coli*. Poultry, cattle, and pork (*C. coli*) isolates were correctly attributed more often than wild bird (*C. jejuni*) isolates (Supplementary table S4). Nearly three-quarters of all human *C. jejuni* cases (n = 8160) were assigned to chicken sources (67.9%; n = 5538), more than a quarter to cattle sources (29.2%; n = 2384), and a small percentage to wild bird sources (2.9%; n = 238) (Fig. 2E, Table 1). Similarly, *C. coli* cases (n = 696) were also pre-dominantly assigned to poultry sources (70.1%; n = 491). However, nearly a fifth of cases were assigned to pork sources (17.1%; n = 119) and only a small amount to cattle (12.4%; n = 86) (Fig. 3E, Table 1). Overall, the majority of campylobacteriosis cases were attributed to poultry (68.1%; n = 6029) and cattle (27.9%; n = 2470), with only a small contribution from wild bird (2.7%; n = 238) and pork sources (1.3%; n = 119; Supplementary table S5). Similar results were obtained using the current best performing core genome MLST (cgMLST) source attribution model (aiSource), which when applied to human cases, also predicted chicken as the primary source of *C. jejuni* infections (Supplementary methods; Supplementary table S6).

### The relative contribution of each infection source reservoir varies between states

Poultry (chicken in *C. jejuni;* chicken and turkey combined in *C. coli*) was identified as the primary source of human campylo-bacteriosis in all 10 HHS regions (Supplementary Fig. S4). The proportion of cases (*C. jejuni* and *C. coli*, combined) attributed to poultry sources ranged from 49% to 87% (Supplementary table S5). In 7/10 HHS regions more than 70% of campylobacteriosis cases could be attributed to poultry sources. This is consistent with other studies that have identified chicken as the primary source of campylo-bacteriosis in the UK, France, Germany, and other countries.^23–26,32,33,34,73^ The lowest proportion of cases attributed to poultry sources was 49% of cases from HHS region 8 (which in-corporates Colorado, Montana, North and South Dakota, Utah, and Wyoming). In this region, a significant proportion of cases were attributed to cattle (49% of *C. jejuni* cases) and pork (26% of *C. coli* cases) sources (Supplementary table S5). According to National Agricultural Statistics Service (NASS) data, this HHS region was not heavily involved in the poultry industry (https://quickstats.nass.usda.gov/; Supplementary table S7).

### Chicken is an increasing source of multidrug resistance Campylobacter in human infections

**Fig. 4:**
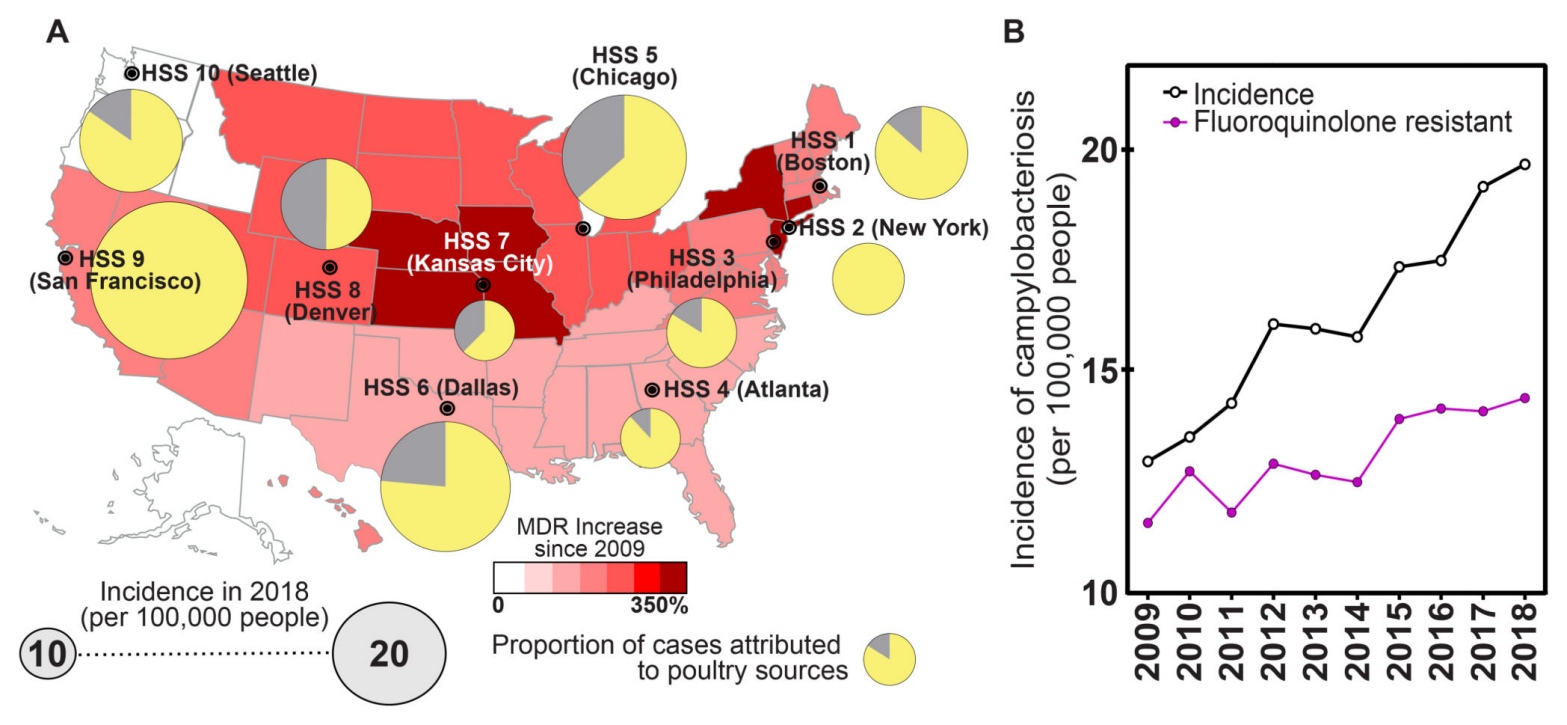
Rise in chicken-associated campylobacteriosis and multi-drug resistance in the US. **(A)** Heat map of the rise in multidrug resistant *Campylobacter* across the US since 2009. Darker red colouring indicates increasing rates of MDR isolates in 2018 compared to 2009. Yellow fractions of pie charts reflect the proportion of isolates from that HSS health region that was predicted to be from chicken sources in our attribution study. Pie chart radius reflects incidence per 100,000 people in 2018 (FoodNet Fast tool: https://wwwn.cdc.gov/foodnetfast/). **(B)** Plot of rising campylobacteriosis incidence (left axis; black line), driven by an increase in the proportion of fluoroquinolone resistant isolates (right axis; purple line).

The general trend over recent years has been for an increasing number of campylobacteriosis cases to be attributed to poultry sources. When combined with incidence rates from the CDC FoodNet Fast tool, the number of infections attributed to poultry sources rose from 22.8% in 2009 to 71.0% in 2018 (Fig. 4A; Supplementary table S5**;** https://wwwn.cdc.gov/foodnetfast/). The poultry-associated clonal complexes, ST-353, ST-443, ST-179, ST-607, ST-508, ST-464, ST-354, ST-460, ST-283 and ST-257 alone accounted for nearly 25% more infections in 2018 than in 2009 (Supplementary table S8). Alongside a documented increase in the incidence of cases, the proportion of multidrug resistant (MDR) isolates (resistance to three or more anti-microbial classes) has also increased since 2009, from 12.3% to 22.3% in 2018 (Fig. 4B; Supplementary table S9); this increase was specifically driven by an increase in fluroquinolone resistance in isolates that infect humans (Fig. 4B; Supplementary table S10).

## Discussion

The global poultry industry rapidly expanded following the Second World War and continues to grow. Chicken is now the primary source of low-cost protein and a staple of the American diet.^76^ Competition in the 1950s transformed the chicken industry, centralising breeding stocks with a limited number of companies.^77^ As a result, the market today is dominated by very few companies that supply breeding stock for the global poultry market, and a single breeder supplies primary stock chickens to more than 130 countries and represents 44% of the global poultry market share.^78^ This rapid expansion in poultry production, alongside a massive decline in chicken diversity, has brought significant challenges in food safety and animal welfare, including infection control.

Addressing these challenges, multiple public health agencies across the US conduct routine surveillance of food-borne pathogens, including *Campylobacter* from clinical infections and potential source populations. While specific clusters of isolates have been linked to outbreak events traced to pets,^13,14^ raw milk,^79^ and chicken livers,^19^ most infections are considered sporadic with no clearly definable point source. Consistent with this, isolates from known outbreaks represented only 0.5% of the cases submitted to FoodNet between 2004 and 2011.^80^ In these circumstances it may be necessary to consider alternative methods for describing disease spread.

Here, by combining genomic data from clinical infections and potential sources, collected by multiple US public health agencies, we were able to assess the relative importance of common food sources for human infection. Most cases in the US were attributed to poultry sources across all HHS regions. This is consistent with previous surveillance efforts of individual states^81^ and European public health agencies.^23,24,30,32,73^ Differences in the relative proportion of cases attributed to poultry have been observed between countries, states, and populations and these have been explained by local demographic (urban v rural)^4^ or dietary^31,33^ differences. However, to date, all national scale source attribution studies have identified poultry as a major reservoir for human infection.

Antimicrobial resistance (AMR) among *Campylobacter* is a concern, not only because this reduces treatment options for serious and persistent infections but also because AMR genes are mobile between strains and species. Widespread fluoroquinolone and macrolide resistance has been reported worldwide^82,83^ and our approach to identifying putative AMR, based on inferred gene function, significantly augments these findings. In particular, isolates belonging to poultry-associated lineages (e.g., ST-353/ST-354 clonal complexes) were found to harbour the most AMR elements.^84^ As clinical isolates reflect populations in source hosts, increasing cases from poultry could increase AMR in human infections. Consistent with this, enteric pathogens from US travellers returning from Asia and South America,^8^ where antibiotic use in livestock is sometimes less regulated,^6^ have high levels of quinolone resistance. The association of AMR with *C. jejuni* and *C. coli* isolates from poultry suggests that even in high-income countries, where AMR is thought to be stabilising,^85^ reducing infection from poultry will have a major impact.

Probabilistic source attribution studies have been successful for assigning *Campylobacter* disease cases to putative source reservoirs.^23,26,30,33,65,86–88^ The fundamental principal is that host adaptation leaves signatures in the *Campylobacter* genome that transcend geographic variation.^42,89,90^ Therefore, when human disease isolates are sequenced, their source can be inferred through genome comparisons. The efficacy of source attribution is largely dependent upon the representativeness of the isolate collection and the specificity of the attribution model. Addressing the need for representative data, our multi-agency cooperative study, combined public health, agricultural, and food regulatory sectors, to create very large-scale surveillance datasets. While this is undoubtedly the largest genomic attribution study to date, it is important to note that US surveillance is heavily weighted towards poultry, beef, and pork (and their source animals) due to perceived risk. Other sources may cause human disease, but this will not impact the primary finding that chicken is the major source. For example, companion animals such as cats and dogs have been implicated as a risk factor for *Campylobacter* infection.^91^ However, their contribution appears to be small, and they may be infected from similar sources to humans.^13,14^

The biology of the bacteria imposes a limit on the specificity of the attribution model. To be informative, genomes used in the model must contain host segregating markers. This requires that the respective isolates have undergone a period of physically isolated (allopatric) evolution in a specific host, long enough to accumulate host-associated genomic signatures. Regular host switching erodes these signatures^74,92^ and it is impossible to attribute an isolate from human infection if an identical strain inhabits two reservoir hosts.^73^ To maximise the identification of host signals, we used machine learning to guide marker selection.^33,65^ In most cases, there was sufficient host segregation to identify host markers. By employing ML methods for marker selection and subsequent attribution, these can be updated in future studies of larger genome collections, with a broader suite of potential infection sources.

Effective public health management is dependent upon evidence to guide interventions and policy. US *Campylobacter* management has included National Advisory Committee (NACMCF) discussions (2007^93^), with minimum standards announced for chickens and turkeys in 2011 and 2016.^94^ Building on these data, our attribution study provides evidence for an increase in the number of cases attributed to poultry sources (since 2011). While there is some difference in attribution between states, this clearly suggests that poultry should be a primary target for reducing the spread of *Campylobacter* and disease incidence with AMR strains. Implementing routine national-scale pathogen genomic surveillance and machine learning source attribution has great utility for monitoring infection trends and supporting interventions to reduce infections by *Campylobacter*, and potentially other pathogens.

## Supplementary Material

**Appendix A. Supporting information**

Supplementary data associated with this article can be found in the online version at doi:10.1016/j.jinf.2024.106265.

supplementary

## Figures and Tables

**Table 1:** Putative source of infection for 8856 campyobacteriosis cases in the US between 2009 and 2019.

	Poultry	Cattle	Pork	Wild bird
*C. jejuni (n = 8160)*	5538 (67.9%)	2384 (29.2%)	–	238 (2.9%)
*C. coli (n = 696)*	491 (70.1%)	86 (12.4%)	119 (17.1%)	–
Total (n = 8856)	6029 (68.1%)	2470 (27.9%)	119 (1.3%)	238 (2.7%)
