# Supplementary material for: Machine learning to attribute the source of Campylobacter infections in the United States: A retrospective analysis of national surveillance data
Source: J Infect. Author manuscript; Available in PMC 2025 Jul 4. (PMC7617841; doi:10.1016/j.jinf.2024.106265)
Supplement: supplementary [file EMS206513-supplement-supplementary.zip › 1-s2.0-S0163445324001993-mmc1.pdf]

Supplementary material for the manuscript:

Machine learning to attribute the source of *Campylobacter* infections in the United States: a retrospective analysis of national surveillance data

Ben Pascoe[1,2], Georgina Futcher[3], Johan Pensar[4], Sion C. Bayliss[5], Evangelos Mourkas[1], Jessica K. Calland[6], Matthew D. Hitchings[7], Lavin A. Joseph[8], Charlotte G. Lane[8], Tiffany Greenlee[9], Nicolas Arning[10], Daniel J. Wilson[10,11], Keith A. Jolley[12], Jukka Corander[6,13,14], Martin C. J. Maiden[12], Craig T. Parker[15], Kerry K. Cooper[16], Erica B. Rose[8], Kelli Hiett[17], Beau B. Bruce[8] & Samuel K. Sheppard[1]#

[1]Ineos Oxford Institute for Antimicrobial Research, Department of Biology, University of Oxford, Oxford, United Kingdom; [2]Pandemic Sciences, University of Oxford, Oxford, United Kingdom; [3]The Milner Centre for Evolution, Department of Biology and Biochemistry, University of Bath, Claverton Down, Bath, United Kingdom; [4]Department of Mathematics, University of Oslo, Oslo, Norway; [5]Bristol Veterinary School, University of Bristol, Langford, Bristol, United Kingdom; [6]Oslo University Hospital, Oslo Centre for Biostatistics and Epidemiology, Oslo, Norway; [7]Swansea University Medical School, Swansea University, Swansea, United Kingdom; [8]Division of Foodborne, Waterborne, and Environmental Diseases, Centers for Disease Control and Prevention, Atlanta, Georgia, USA; [9]Center for Food Safety and Applied Nutrition, Food and Drug Administration, College Park, Maryland, USA; [10]Big Data Institute, Oxford Population Health, University of Oxford, Li Ka Shing Centre for Health Information and Discovery, Old Road Campus, Oxford, United Kingdom; [11]Department for Continuing Education, University of Oxford, United Kingdom; [12]Department of Biology, University of Oxford, Oxford, United Kingdom; [13]Department of Mathematics and Statistics, University of Helsinki, Helsinki, Finland; [14]Parasites and Microbes, Wellcome Sanger Institute, Cambridge, United Kingdom; [15]Produce Safety and Microbiology Research Unit, Agricultural Research Service, US Department of Agriculture, Albany, California, USA; [16]School of Animal and Comparative Biomedical Sciences, University of Arizona, Tucson, Arizona, USA; [17]Center for Food Safety and Applied Nutrition, Food and Drug Administration, Laurel, Maryland, USA.

#Author to which correspondence should be addressed:
Samuel K Sheppard, University of Oxford, Oxford, UK
samuel.sheppard@biology.ox.ac.uk

Table of contents

3 **Figure S1**: Overview of Health and Human Services regions.
4 **Figure S2**: Workflow schematic.
5 **Figure S3**: Summary of main sequence types identified in clinical isolates.
6 **Figure S4**: Summary of attribution analysis results.

[separate .xlsx file]
Table S1: Genomes from US campylobacteriosis cases between 2009-2019.
Table S2: Genomes from non-human contextual isolates from possible infection sources.
Table S3: Summary table of host segregating markers.
Table S4: Asymmetric Island self-test summary.
Table S5: Summary of analysis to attribute the source of clinical isolates.
Table S6: Summary of analysis with cgMLST using aiSource.
Table S7: Correlation of attribution results with publicly available demographic data.
Table S8: Breakdown of attributed human clinical cases by ST and year.
Table S9: Proportion of human clinical isolates that are multidrug resistant.
Table S10: Proportion of human clinical isolates that are fluoroquinolone resistant.

Additional materials and files

Figshare: All supplementary files can also be found on FigShare doi: 10.6084/m9.figshare.20279928

Mendeley: Contiguous assemblies of all genome sequences compared are available at Mendeley data.
C. coli doi: 10.17632/gxswjvxyh3.1
C. jejuni doi: 10.17632/6ngsz3dtbd.1

Microreact: Interactive phylogenies are hosted separately for
C. jejuni: https://microreact.org/project/pascoe-us-cjejuni
C. coli: https://microreact.org/project/pascoe-us-ccoli

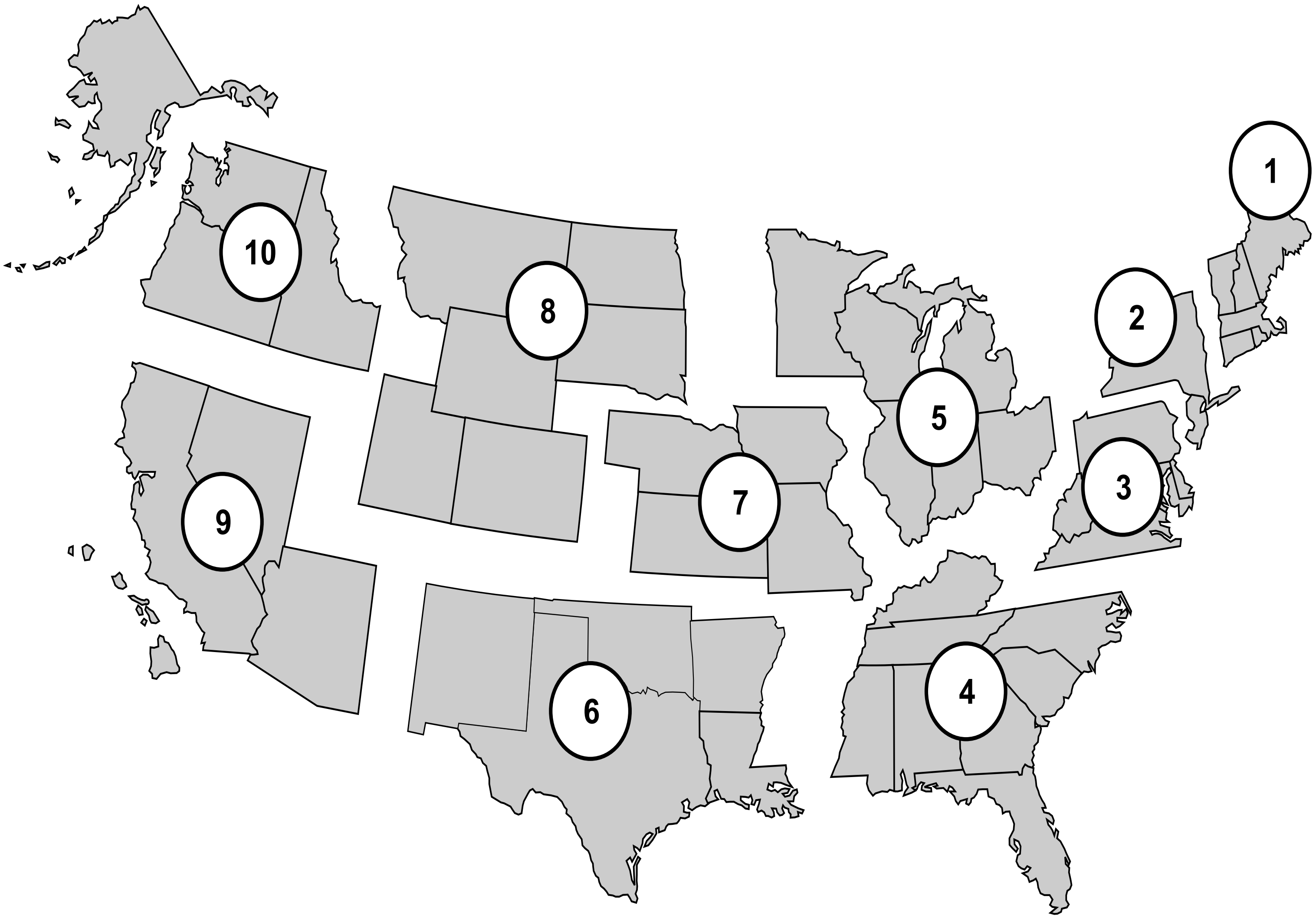

Supplementary figure S1

Samples were collected from suspected campylobacteriosis cases and reported nationally by ten regional offices: **Region 1** (Boston) includes Connecticut, Maine, Massachusetts, New Hampshire, Rhode Island and Vermont; **Region 2** (New York): New Jersey, New York, Puerto Rico and the Virgin Islands; **Region 3** (Philadelphia): Delaware, District of Columbia, Maryland, Pennsylvania, Virginia, and West Virginia; **Region 4** (Atlanta): Alabama, Florida, Georgia, Kentucky, Mississippi, North Carolina, South Carolina, and Tennessee; **Region 5** (Chicago): Illinois, Indiana, Michigan, Minnesota, Ohio, and Wisconsin; **Region 6** (Dallas): Arkansas, Louisiana, New Mexico, Oklahoma and Texas; **Region 7** (Kansas City): Iowa, Kansas, Missouri and Nebraska; **Region 8** (Denver): Colorado, Montana, North Dakota, South Dakota, Utah, and Wyoming; **Region 9** (San Francisco): Arizona, California, Hawaii, Nevada, American Samoa, Commonwealth of the Northern Mariana Islands, Federated States of Micronesia, Guam, Marshall Islands, and Republic of Palau; **Region 10** (Seattle): Alaska, Idaho, Oregon and Washington.

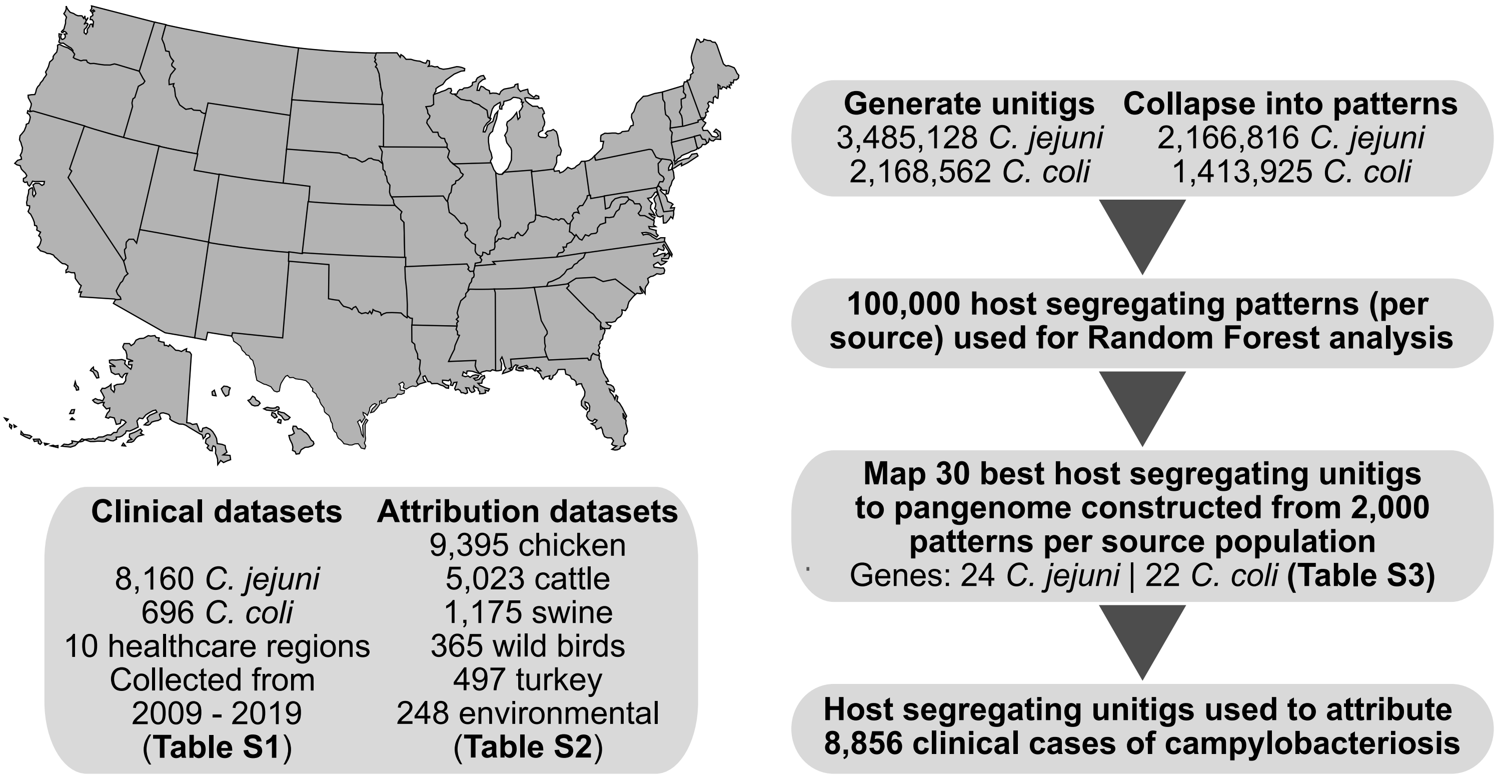

Supplementary figure S2

Schematic overview of analysis pipeline. Isolates were sampled from potential sources and from human infections. In total, a collection of over 25,000 genomes were used in the analysis (18,333 *C. jejuni* and 7,226 *C. coli*). Genomes were fragmented into unitgs and grouped into presence-absence patterns of unitigs found in the same subset of isolates. The distribution of patterns between sources was scored according to their association with a given source and 100,000 (per source) were selected for random forest analysis. Patterns of untigs were then mapped back to a pangenome to identify genes containg the most (5 per source) and strongest (highest MI score) associated unitigs. These 30 unitigs per species (24 *C. jejuni* and 22 *C. coli* genes) were used as our host-segregating markers. Turkey and chicken were combined as a single source for attribution analyses with *C. coli*, as they could not be accurately segregated. iSource and aiSource algorithms were run 10 times and self-attribution was performed for each species, masking the source of one third of isolates from non-clinical populations. Clinical isolates were attributed separately for each species.

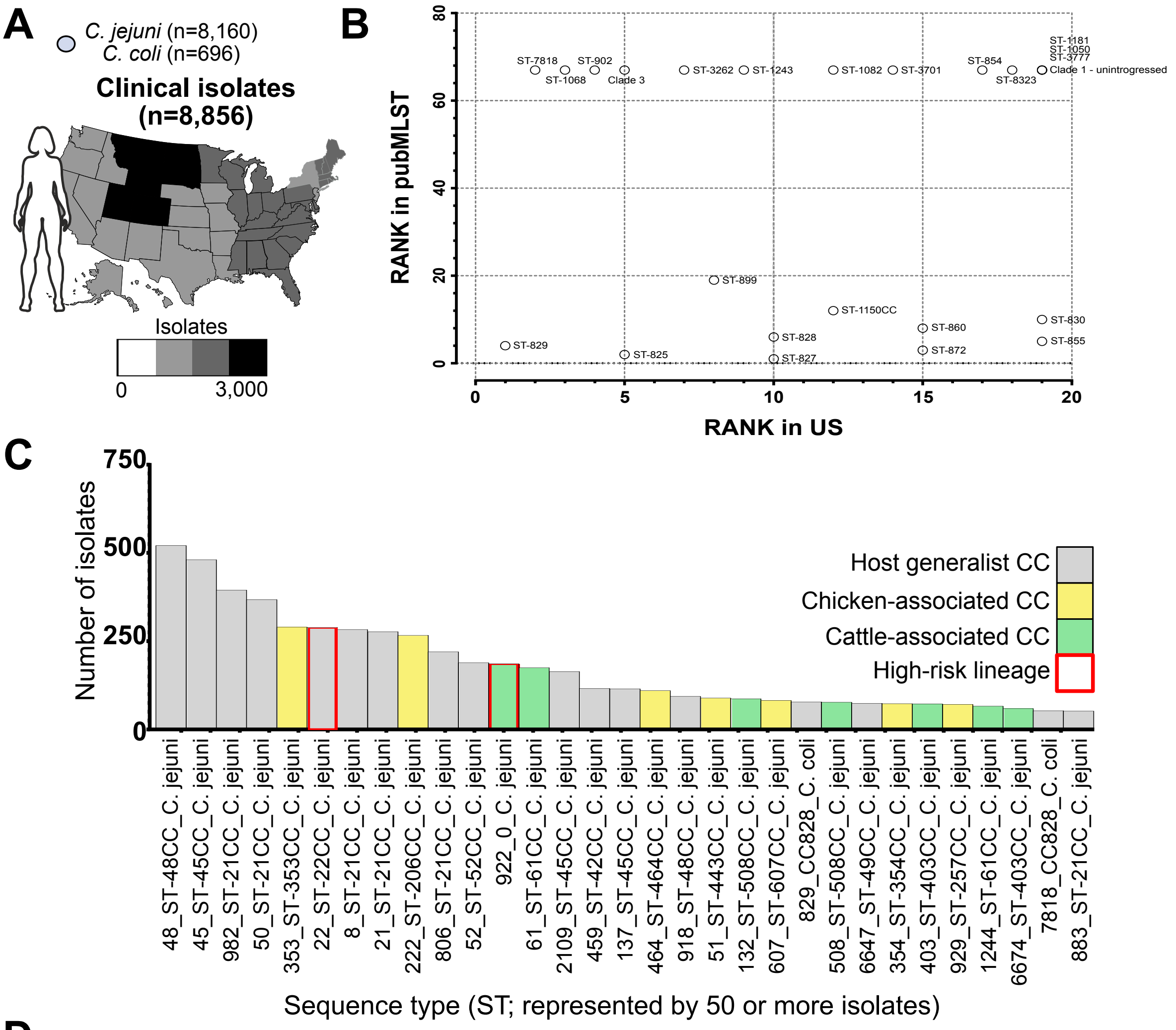

D

	Most common	2	3	4	5
**HSS 1**	ST45_(CC45)	ST353_(CC353)	ST48_(CC48)	ST50_(CC21)	ST52_(CC52)
**HSS 2**	ST45_(CC45)	ST2109_(CC45)	ST50_(CC21)	ST922_(CC922)	ST354_(CC354)
**HSS 3**	ST50_(CC21)	ST45_(CC45)	ST48_(CC48)	ST22_(CC22)	ST353_(CC353)
**HSS 4**	ST48_(CC48)	ST353_(CC353)	ST50_(CC21)	ST222_(CC206)	ST464_(CC464)
**HSS 5**	ST8_(CC21)	ST48_(CC48)	ST982_(CC21)	ST21_(CC21)	ST50_(CC21)
**HSS 6**	ST48_(CC48)	ST222_(CC206)	ST22_(CC22)	ST353_(CC353)	ST982_(CC21)
**HSS 7**	ST2109_(CC45)	ST48_(CC48)	ST21_(CC21)	ST45_(CC45)	ST61_(CC61)
**HSS 8**	ST982_(CC21)	ST45_(CC45)	ST806_(CC21)	ST48_(CC48)	ST8_(CC21)
**HSS 9**	ST8_(CC21)	ST52_(CC52)	ST222_(CC206)	ST22_(CC22)	ST51_(CC443)
**HSS 10**	ST48_(CC48)	ST45_(CC45)	ST22_(CC22)	ST222_(CC206)	ST50_(CC21)

Supplementary figure S3: Summary of clinical isolates collected. **(A)** *C. jejuni* (n=8,160) and *C. coli* (n=696) isolates were collected from across the US (total: 8,856). **(B)** Distribution of common ST clonal complexes, compared to global collections (pubMLST). **(C)** Number of isolates collected from all ST clonal complexes represented by 50 or more isolates. Coloured by previously published associations with specific food sources. **(D)** Most common clonal complexes, by individual health region.

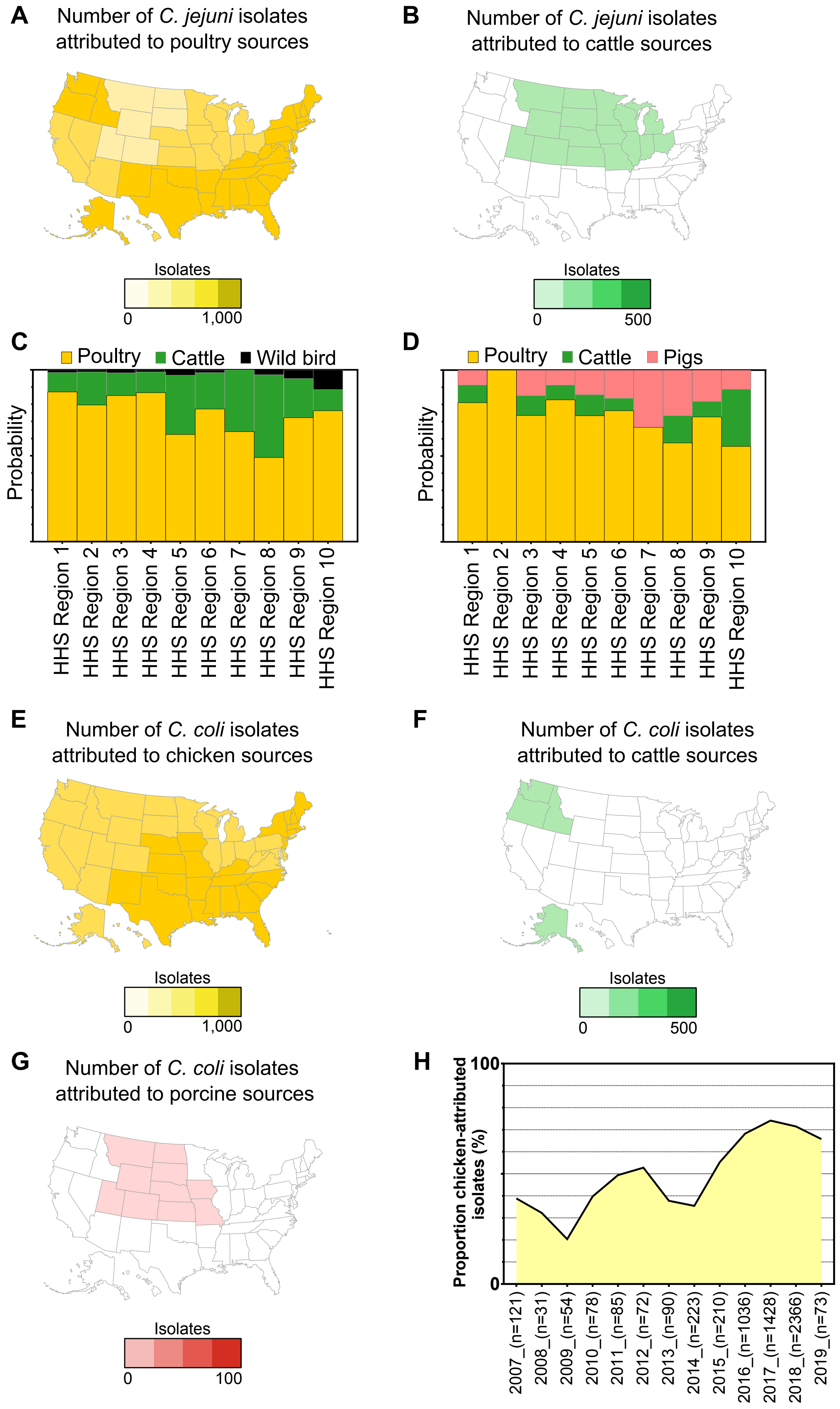

Supplementary figure S4: Attribution of US clinical isolates with iSource using host segregating markers. Maps of the US health regions, coloured by the number of *C. jejuni* isolates attributed to **(A)** poultry and **(B)** cattle sources. Bar chart breakdown of each health region for **(C)** *C. jejuni* and **(D)** *C. coli* isolates. Maps of the US health regions, coloured by the number of *C. coli* isolates attributed to **(E)** poultry, **(F)** cattle and **(G)** pork sources. **(H)** Trend towards inreased attribution of total numbers of isolates to poultry sources, from 2007 to 2019.
